# Management of the Malignant Rectal Polyp—A Narrative Review

**DOI:** 10.3390/cancers17091464

**Published:** 2025-04-27

**Authors:** Zhen Hao Ang, Shing Wai Wong

**Affiliations:** 1 Department of Colorectal Surgery, Prince of Wales Hospital, Sydney, NSW 2031, Australia; sw.wong@unsw.edu.au; 2 Randwick Campus, School of Clinical Medicine, University of New South Wales, Sydney, NSW 2033, Australia

**Keywords:** malignant polyp, early colorectal cancer, management

## Abstract

Purpose: The aim of this review is to provide a contemporary update on the current management approaches and options with specific considerations in malignant rectal polyps. Methods: A literature review was carried out in PubMed, Embase and Cochrane databases using the keywords “malignant” and “polyp*”. Only publications in English language were included. Results: Histopathological features including margins, depth of invasion, tumour grade, LVI and tumour budding determines the risk of lymph node metastasis in malignant polyps. Rectal malignant polyps should be considered differently compared to their colonic counterpart. A low threshold should be considered for utilising transrectal excision to fully excise the polyp and to assess the margins. The rates of complete pathological response associated with total neoadjuvant therapy as well as the advent of “watch and wait” adds to the complexity of managing malignant rectal polyps. Conclusions: The management of malignant colorectal polyps lies in risk-stratifying patients who will benefit from an oncological resection.

## Simple Summary

Malignant polyps are endoscopically resected polyps which harbour a focus of carcinoma on histological examination. These lesions are most diagnosed post-polypectomy. Management of these lesions can be complicated based on the histopathological characteristics, risk of recurrence, as well as the location of the lesion. Newer techniques and the evolution of neoadjuvant and adjuvant therapy adds complexity to the management algorithm.

## 1. Introduction

Colorectal cancer (CRC) is the leading cause of cancer-related deaths and the third most common cancer worldwide. It accounts for about 10% of all cancer cases and is considered a major public health issue globally [1]. Most CRC develops from adenomatous polyps. A polyp is an abnormal protrusion from the colonic mucosal surface. It is widely accepted that these polyps gradually grow for 10–20 years before becoming cancerous (adenoma–carcinoma sequence). Adenomatous polyposis Coli (APC) gene mutation and RAS mutation have been implicated in the formation of colorectal polyps. The three main pathways involved in CRC are the chromosomal instability pathway, the microsatellite instability (MSI) pathway and the CpG island hypermethylation pathway. The vast majority of CRC is sporadic (70%), with hereditary conditions only responsible for 5% of cases. The main risk factors for CRC include obesity, red meat, processed meat, a diet high in sugar and animal fat as well as alcohol.

Surgery, with adequate margins and lymph node (LN) harvest, remains the primary treatment with curative intent. Other modes of treatment, including chemotherapy, radiotherapy and immunotherapy, are either used as an adjunct or in the palliative setting [2].

Malignant polyps (MPs) are a special subgroup of CRC. Malignant polyps are endoscopically resected polyps which harbour a focus of carcinoma on histological examination. The focus of the cancer in these polyps invades into the submucosa but does not extend into muscularis propria [3]. They are classified as pT1 in the American Joint Committee on Cancer (AJCC) 8th edition TNM staging classification for CRC [3]. Depending on series, malignant polyps account for 1–12% of colorectal polyps removed during colonoscopy [4,5].

Malignant polyps represent the earliest form of clinically significant CRC as the invasion into the submucosa means that lymphovascular invasion is possible. As a result, the subsequent management following endoscopic resection of malignant polyps depends on the risk of lymphatic spread and the need for an oncological resection [3]. The aim of this review is to provide a contemporary review of the current understanding and evidence in the management of malignant polyps with specific attention to malignant rectal polyps. In this review, we will first be discussing endoscopic diagnosis of malignant polyps, followed by a discussion on the management of malignant colorectal polyps in general, and finally how malignant rectal polyps are different from their colonic counterpart.

## 2. Method

A literature review was carried out in the PubMed, Embase and Cochrane databases using the keywords “malignant” and “polyp*”. Search was limited to articles published in English and were published from year 2000 to capture the most contemporary evidence. Forward search and review of references was also carried out on shortlisted articles to identify other relevant publications missed in the initial search.

## 3. Discussion

### 3.1. Endoscopic Features Suggestive of Polyps Harbouring Malignancy with Submucosal Invasion

The morphology of polyps can be classified into pedunculated or non-pedunculated (sessile). Sessile polyps lack the protective stalk of pedunculated polyps and are more likely to harbour lymphatic invasion at time of diagnosis.

Polyp size is closely related to the likelihood of the polyp harbouring malignancy. In the reported series, no polyps < 5 mm in diameter contained any malignant component. In the same series, polyps greater than 2.5 cm in diameter have a close to 50% chance of harbouring malignancy [6]. Size and morphology should hence be considered when estimating the risks of malignancy in a polyp [7].

**Figure 1:**
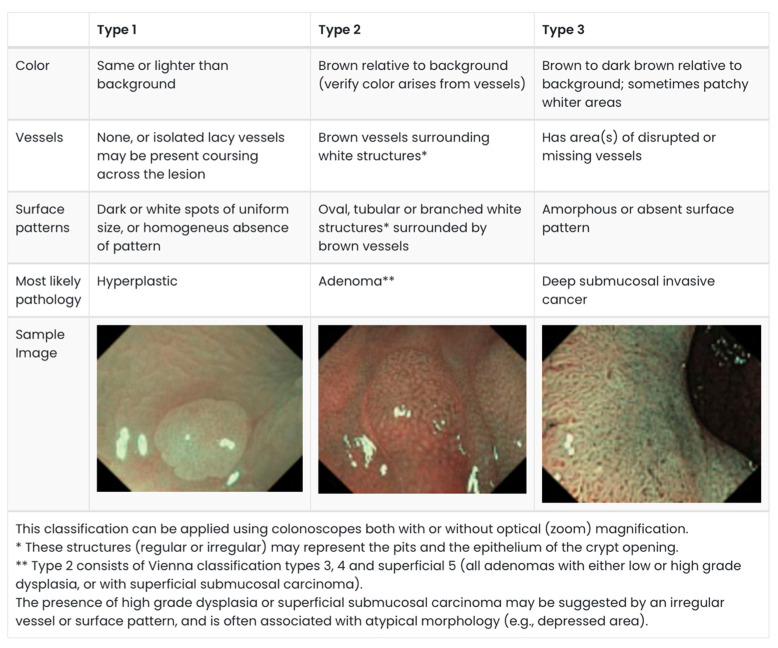
NICE Classification. Adapted from: Endoscopy Centre; polyp classification: NICE [10,12].

Apart from the size and morphology, several endoscopic surface-pattern classifications have also been described with the aim of predicting malignancy, specifically submucosal invasion. The Narrow-Band imaging International Colorectal Endoscopic (NICE) classification is a well-validated system for classifying polyps. This system categories polyps into three categories based on their appearance on Narrow-band Imaging (NBI), with Type 3 polyps concerning for submucosal invasion (Figure 1) [8,9,10]. Using NBI ameliorates the need for dye spray.

**Figure 2:**
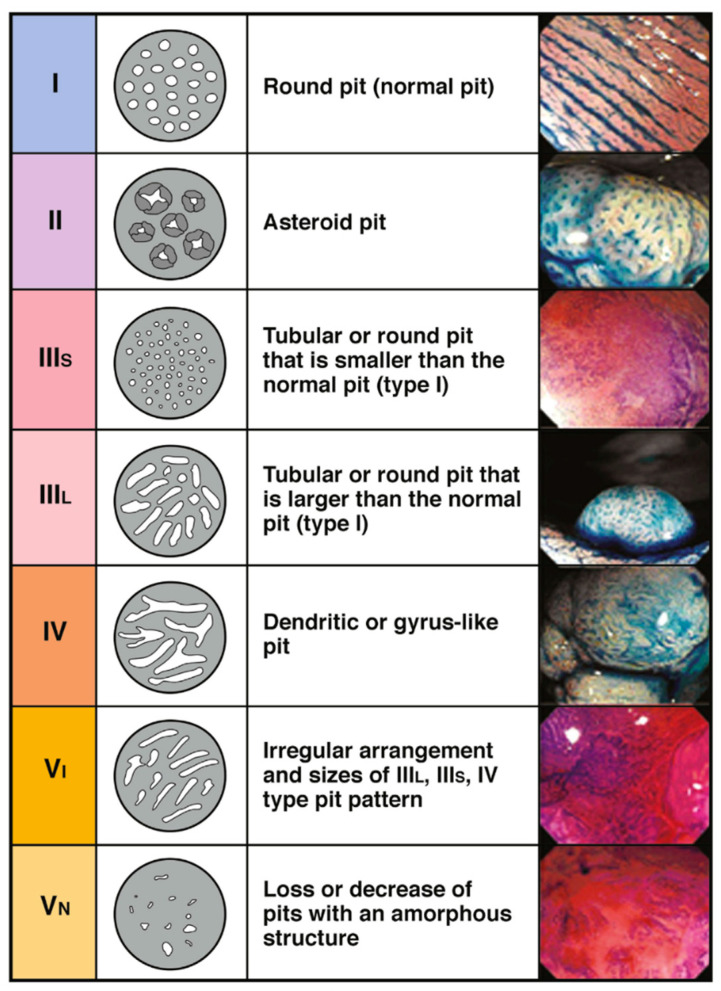
Kudos pit pattern. Adapted from Shaukat et al., 2020 [3].

Another commonly utilized system is the Kudo’s pit pattern classification which separates the pit patterns into six classifications (Figure 2), with Type III–V suggestive of dysplastic and malignant transformation [11]. However, the Kudos system was originally described using dye spray to accentuate the pit patterns.

The presence of “non-lifting sign” was also described by Uno et al. as a way of determining submucosal involvement (either by malignancy or fibrosis). To elicit this sign, fluid is injected in the submucosal plane under the polyp, and it is considered present when there is failure to lift the polyp [13].

### 3.2. Initial Approach to Possible Malignant Polyps

Endoscopic management of suspicious pedunculated polyps are more favourable compared to sessile polyps. The presence of a stalk makes complete resection more likely, hence attempts should be made to endoscopically resect these polyps en bloc [3].

For non-pedunculated lesions, more careful consideration is required. A study found that lesions larger than 20 mm with Kudo’s pit pattern V were strongly associated with overt submucosal cancer, with a specificity of 97% and diagnostic accuracy of 93% [14]. A meta-analysis by Li et al. also reported that applying Kudo’s pit pattern achieved a pooled sensitivity of 89% and specificity of 85.7% in predicting invasive lesions [15]. This led to a recommendation by the American Gastroenterological Association (AGA) that non-pedunculated polyps with NICE 3 or Kudo’s pit pattern V be biopsied and SPOT marked rather than endoscopically resected [3]. The current consensus is that if a polyp (especially large polyps) is suspected to be malignant, endoscopic resection should not be attempted unless it can be resected en bloc [16].

### 3.3. Initial Approach to Histologically Proven Malignant Polyps

The key importance of histological features in endoscopically resected malignant polyp lies in predicting the risk of locoregional lymph node metastasis (LNM). Studies that attempted to delineate these high-risk features were derived from resected specimens [3]. Systematic reviews evaluating the risks of LNM in malignant polyps consistently reported submucosal invasion >1 mm (resection margin <1 mm for pedunculated polyps), tumour budding, presence of lymphovascular invasion and poorly differentiated histology as high-risk features for LNM [17,18]. It is important to note that whilst depth of invasion is an important high-risk feature in non-pedunculated malignant polyps, resection margin of <1 mm is more relevant in pedunculated malignant polyps.

#### 3.3.1. Depth of Invasion and Margin

The two main histological classifications of malignant polyps are dependent on their macroscopic appearances. Both attempt to classify the depth of invasion of the malignant component within a polyp, hence predicting the likelihood of lymphatic invasion. The Haggitt classification [19] is typically used in malignant pedunculated polyps and the Kikuchi classification [20] is used for sessile polyps.

**Figure 3:**
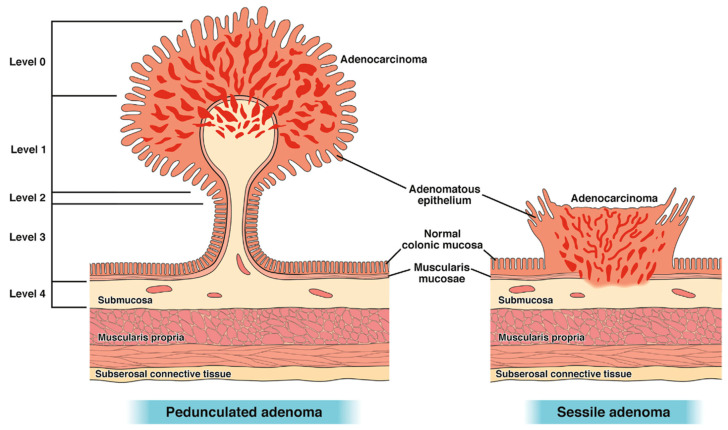
Haggitt classification. Adapted from Shaukat et al., 2020 [3].

Haggitt et al. described invasion from levels 0–4, with levels 1–4 having submucosal invasion (Figure 3). Haggitt 1 polyps have submucosal invasion limited to the head of the polyp while levels 2 and 3 involve the neck and stalk, respectively. Haggitt 4 polyps have submucosal invasion below the stalk. All sessile polyps are Haggitt 4 by definition (without a stalk) [19].

**Figure 4:**
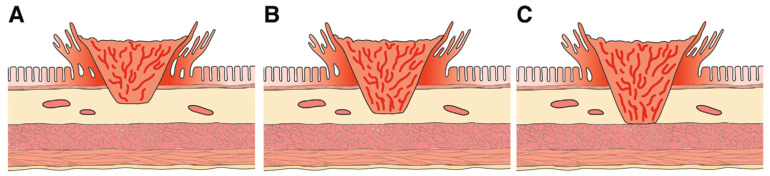
Kikuchi classification. (**A**) SM1; (**B**) SM2; (**C**) SM3. Adapted from Shaukat et al., 2020 [3].

For malignant sessile polyps, the Kikuchi classification separates these polyps into three levels of submucosal invasion [20]. SM1 invasion denotes invasion into the upper third of the submucosal layer and SM2 and SM3 denote invasion into the middle and deepest third, respectively (Figure 4). The difficulty with implementing Kikuchi in endoscopically resected specimens stems from the rarity of the entire submucosa being included in the specimen (i.e., visualization/presence of muscularis propria) [3]. For this reason, pathologists largely report the depth of invasion itself. Depth of invasion < 1 mm is considered superficial, with a low risk of LNM (0–4%). Depth of invasion of 1mm and more is considered deep, with risk of residual malignancy and LNM of 10–18% [21].

Kikuchi SM1 depth of invasion was associated with up to 3% risk of LNM, which rises to 8% and 23% for SM2 and SM3, respectively [20]. As discussed previously, the main drawback with the Kikuchi level is the general absence of muscularis propria within the specimen, hence making the assessment of the entire submucosa not possible. As a result, the depth of invasion in non-pedunculated polyps is divided into superficial (<1 mm) and deep (>1 mm), with the risk of LNM rising with increased depth. Depth of invasion of <1 mm is widely regarded as having negligible risk of LNM with a multi-centre Japanese study reporting a 0% LNM rate [22]. Beaton et al. reported in their meta-analysis of 23 cohort studies that a depth of 1–2 mm is associated with a 1.3–4% risk of LNM [23]. This rises to 12–18% when the depth of invasion is >2mm. This is consistent with another systematic review by Choi et al. which reported that deep invasion was associated with three times higher risk of LNM [21].

For pedunculated polyps, studies have reported Haggitt 4 polyps to be at high risk of LNM (27% prevalence compared to 0% in one study when compared to Haggitt 1–3 polyps) [3,24]. In clinical practice, transection of the polyp often occurs at the stalk and not below it, hence the clearance margin is often used as a surrogate to differentiate Haggitt 3 from 4. Studies have found that a margin of >1mm was associated with a reduced local recurrence and LNM (2% vs. 33%) [5,25]. An indeterminate margin should also be considered a high-risk feature [26].

#### 3.3.2. Tumour Grade

Poorly differentiated adenocarcinoma is rare in malignant polyps [7]. However, poor differentiation is a strong predictor for LNM. Studies have reported rates of LNM ranging from 25 to 86% [23].

#### 3.3.3. Lymphovascular Invasion

Reviews and individual studies have identified lymphovascular invasion (LVI) as an independent risk factor for LNM with up to 45% of patients with LVI having LNM reported in one study [3,27]. A meta-analysis of 31 studies by Hassan et al. found that the presence of LVI is associated with a higher risk of LNM with an odds ratio (OR) of 7 found within the pooled analysis [28]. Beaton et al. reported rates of LNM of 15–82% in their meta-analysis [23].

#### 3.3.4. Tumour Budding

Tumour budding is defined as the presence of a single cell or a cell cluster of four or fewer tumour cells at the invasive margin of the polyp. Previous meta-analysis found tumour budding to be associated with a 26–64% chance of LNM [23]. A recent Chinese study also reported tumour budding to be present in 42% of specimens with LNM compared to 18% in patients without LNM (OR 2.3) [29]. Therefore, it is crucial for pathologists to detail these findings within the report to allow risk stratification and decision regarding formal oncological resection versus endoscopic surveillance. The National Comprehensive Cancer Network (NCCN) guidelines also recommended surgery for malignant polyps harbouring the high-risk features reported above [30].

### 3.4. Management of Histology Proven Malignant Polyps

#### 3.4.1. Staging

Being early cancers, patients with malignant polyps should be staged similarly to all CRCs. Patients should have computed tomography (CT) scans of the thorax, abdomen and pelvis. Tumour marker (CEA) should be performed in all patients. Patients with malignant rectal polyps should have a magnetic resonance imaging (MRI) of the rectum to complete local staging [30].

#### 3.4.2. Is Endoscopic Resection Sufficient?

The key difference between an endoscopically resected malignant polyp versus a surgically resected oncological specimen is the ability of the latter approach to assess the adjacent draining lymph nodes.

A retrospective population-based study utilizing the Surveillance, Epidemiology, and End Results (SEER) database found that patients who underwent surgical resection for malignant polyps had a better 1- and 5-year survival rate compared to those with polypectomy alone. When adjusted for co-morbidities, age and histology (only tumour grade was considered), no statistical difference was found in terms of survival.

This further supports the management of non-piecemeal endoscopically resected malignant polyps with clear margins (>1 mm) and in the absence of high-risk features (as described in the previous section), with surveillance and not rectal resection [7]. Dang et al. reported in their meta-analysis of 71 studies that about 3.3% of endoscopically resected pT1 CRC recur either locally or systemically within 6 years [31]. In low risk pT1 CRC, recurrence occurred in 0.7% of cases as opposed to 7% in high-risk pT1 CRC. Moreover, recurrences in the low-risk group typically recur locally [31].

#### 3.4.3. Piecemeal Resected Malignant Polyps

If the initial polypectomy was performed piecemeal, the decision making becomes more challenging. Recurrence or persistent polyps (all polyp morphology) have been found to occur in 14% from pooled data from a meta-analysis [28]. This likely reflects the difficulty faced by the endoscopist in identifying the macroscopic margins of the polyp. The pathologist faces similar challenges with the microscopic margin as it is impossible to piece the fragments together to clearly delineate each margin [7].

Gibson et al.’s study reported the oncological outcomes of covert malignant non-pedunculated polyps which were resected piecemeal [16]. In their study, they reported a high rate of residual tumour found in surgically resected specimens (37%) compared to the meta-analysis of Hassan et al. (33%) [28]. However, they acknowledged that this higher rate could be due to their cohort including only large non-pedunculated polyps, which itself carries a higher risk of deeper submucosal involvement. Importantly, in their cohort of patients with residual malignancy found post-resection, almost all of them had involved margins on piecemeal histology., Patients who had well or moderately differentiated tumour and no LVI did not have LNM. Whilst this study alone does not confer universal guidance, it does provide the best available evidence when discussing surgery versus surveillance for patients who had covert malignant polyps resected piecemeal [16].

The current guidelines recommend careful patient counselling and discussion with a low threshold for surgical resection [30]. Patients with malignant polyps with high-risk features should be counselled for surgical resection.

#### 3.4.4. Role of Biomarkers in Decision Making

Biomarkers in CRC play a key role in its diagnosis and influences management. Biomarkers in cancer can be categorised into diagnostic, predictive and prognostic. Prognostic biomarkers play a key role in influencing treatment as it helps delineate patients with high-risk pathology, who will benefit from more aggressive treatment, from those with low-risk pathology [32]. In CRC, these biomarkers can be categorised in four consensus molecular subtypes (CMS) groups with varying prognostic implications (Table 1) [33]. The presence of tumour-infiltrating lymphocytes (TIL) has also been associated with a better prognosis in patients with CRC [33].

A multi-centre Dutch study found that in T1 CRC, MSI-high (MSI-H) tumours and tumours with high TIL were associated lower LNM. Conversely, in the CMS4 group, risk of LNM and recurrent cancer was much higher (66.7% 5-year event-free survival compared to 87.5% in CMS1/2/3) [34]. This was consistent with Sugai et al. who reported that mesenchymal-like markers were correlated with LNM in T1 CRC [35]. A Korean study by Kang et al. further supported the low risk of LMN associated with MSI-H T1 tumour [36].

At present, apart from MSI, the costs associated with performing these biomarkers routinely for all malignant polyps is likely prohibitive. The sensitivity and specificity of these biomarkers in the prediction of LNM will likely still require validation from large studies to be validated.

#### 3.4.5. Surveillance

Whilst there is no clearly specified surveillance interval, authors have recommended repeating colonoscopy 6–12 months after endoscopically resected malignant polyps [7,31]. In high-risk groups who did not undergo surgical resection, surveillance should include both endoscopic and cross-sectional imaging (for distant metastasis) [31].

### 3.5. Role of Surgery

**Figure 5:**
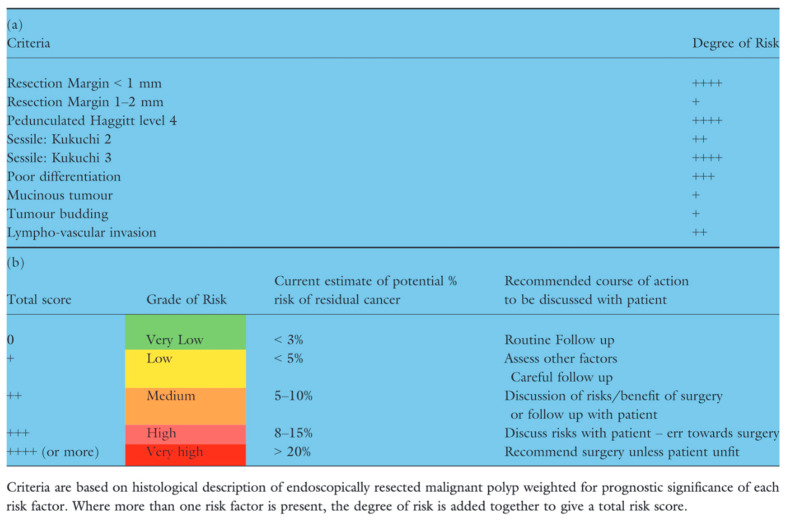
ACPGBI risk stratification tool. Adapted from Williams et al., 2013. (**a**) represents the high-risk features and (**b**) represents the risk of malignancy [4].

The Association of Coloproctology of Great Britain and Ireland (ACPGBI) guidelines for the management of malignant polyps not only summarises the high-risk features of locoregional recurrence but also provides a risk stratification to guide clinicians in management and patient counselling. It classifies the risk in five categories from very low to very high (Figure 5).

Despite the presence of the guidelines, large population studies suggest heterogeneity in adherence. Zammit et al. performed an Australian-based population analysis of 1646 malignant polyps and found that 31.6% of very low- or low-risk patients went on to have surgical resection and 36.7% of high- and very high-risk patients did not have surgery [37]. The study also found that younger patients (with their perceived lower surgical risk and longer life expectancy) and patients with right-sided malignant polyps (with right-sided resections being potentially technically easier and associated with less morbidity) were more likely to proceed to surgery. This is compared to patients with left-sided malignant polyps (with potentially easier sigmoidoscopy surveillance as opposed to the need for full bowel preparation to perform colonoscopy). However, the American Society of Anaesthesiologist (ASA) score was not significantly different between the groups that went on to have formal resection versus surveillance, which suggests that the ASA score did not play a key factor in deciding between surgery versus surveillance [37]. These findings were similar in a Scottish observational study by Johnstone et al. [38].

A survey of Australian subspecialty colorectal surgeons also reflected higher tolerance for risk in left-sided malignant polyps—twice that of the 5% risk of recurrence or LNM that was widely accepted [39].

These factors highlight that whilst high-risk features inform risks of recurrence and LNM, the final decision regarding surgical resection remains a complex interplay of what is an acceptable risk to the surgeon and the patient.

### 3.6. Malignant Rectal Polyps

#### 3.6.1. Introduction to Malignant Rectal Polyps

Due to the differing regimens in the management of sigmoid and rectal cancers, it is important for clinicians to clearly define where the rectum is. Considerable variation in the length of the rectum and the anal canal makes endoscopic identification of the upper rectum (which is based on distance from the anal verge) inaccurate. Hence, the use of radiological and anatomical definition provides more consistency [40]. Radiologically, the upper border of the rectum lies between the S1–S3 vertebral levels, and in a recent Delphi consensus, starts at the “sigmoid take-off” (which is the recto-sigmoid junction) [41]. Intra-operatively, the start of the rectum is determined by the convergence of tinea coli, the onset of the peritoneal reflection and the level of the sacral promontory [40].

Morphologically, rectal polyps are more likely to be sessile than pedunculated [42]. Unlike in colon cancer, neoadjuvant chemoradiotherapy plays a crucial role in reducing local recurrence rates in rectal cancer. With the growing evidence behind total neoadjuvant therapy and its superior complete pathological response rates, there is a push for rectal preservation with the watch and wait (W&W) approach [7]. Population studies have also showed that rates of LNM are higher in rectal cancers compared to colon cancers with matching T-stage [43].

Nusko et al. also reported that polyps found in the rectum have a higher risk of containing invasive carcinoma compared to its colonic counterparts [6].

#### 3.6.2. Management Approach

##### Role of Imaging

Imaging plays a key role in the management of the malignant rectal polyp, specifically for local staging [44]. With improved technology, the better resolution of rectal MRI studies allows for a more precise delineation of the layers of the rectum. However, clinicopathological studies have found MRI to be not completely reliable in distinguishing T1 from T2 rectal cancers [45,46]. However, MRI does allow assessment of the locoregional lymph nodes as well as presence of tumour deposits and extramural venous invasion, which are all predictors of more advanced disease [47]. In a post-polypectomy setting, inflammatory or artifactual changes further limit the accuracy of T and N staging by MRI study [48].

The role of endorectal ultrasound (ERUS) in local staging for rectal malignancy has taken a back seat due to the accessibility of MRI and the inability to assess the mesorectal lymph nodes reliably. It is important to note that despite this, ERUS still remains a sensitive and specific tool in delineating early cancers and may be more reliable in distinguishing T1 and T2 rectal cancers [49].

Positron emission tomography (PET) has a well-established role in distant metastatic CRC and is recommended by major guidelines for this subset of patients. In the assessment of lymph nodes, it complements the size and morphological criteria used in MRI and conventional CT [50]. A Swiss retrospective cohort study reported that PET conferred improved sensitivity and specificity when compared to CT alone for nodal assessment. A similar improvement was not observed when compared to rectal MRI alone [50]. Major guidelines such as the NCCN guidelines recommend against using PET scans as part of initial staging for non-metastatic CRC [30].

### 3.7. Role of Multi-Disciplinary Team (MDT) Meeting in Decision Making

The role of MDT in the management of CRC is well established. However, its role in the management of the malignant rectal polyp has not been investigated. Parallels can be drawn from the experience of MDT in the management of more advanced CRC. A well-run MDT provides a patient-centred approach to the management of each case of a malignant polyp [51]. An experienced gastrointestinal pathologist and radiologist play an important role in the management of malignant rectal polyps, as the histological findings and MRI findings dictate management [7]. Drawing experiences from stage II and III rectal cancers, MDT discussions have been demonstrated as resulting in improvements in disease-free survival compared with no MDT input [51].

### 3.8. Does Height of Malignant Rectal Polyp Matter?

Venous drainage in the rectum, unlike the colon, is varied depending on the location. It has been shown that upper rectal tumours are more likely to metastasize to the liver (like the colon), whereas pulmonary metastasis is more common in lower rectal tumours. This is believed to be due to the middle and inferior rectal venous systems draining into the systemic circulation as opposed to the portal venous system in the upper rectum [52].

Moreover, studies have reported higher rates of LNM and local recurrence after local excision of low rectal T1 cancers compared with upper rectal T1 cancers [53].

#### 3.8.1. Surgical Options for Malignant Rectal Polyps

Three different treatment modalities are available for the management of malignant rectal polyps. Options include endoscopic polypectomy, trans-anal resection (TAR) and rectal resection with total mesorectal excision (TME) [54].

#### 3.8.2. EMR/ESD

Endoscopic approaches include snare polypectomy, endoscopic submucosal resection (EMR) and endoscopic submucosal dissection (ESD) techniques. Snare polypectomy remains the most common method for removing polyps found during endoscopy. This is a safe technique that is easily replicable and effective for small polyps [4]. EMR is seen as an extension to conventional polypectomy where gelfusine is injected into the submucosal plane to lift the polyp off the underlying muscularis propria. This allows the en bloc resection of sessile polyps with a more intact deep margin [55]. ESD, on the other hand, not only involves lifting the polyp off the muscularis, but the polyp is also resected using an endoscopic knife. This technique allows larger polyps to be resected endoscopically whilst maintaining an adequate lateral and deep margin. This technique typically requires a trained endoscopist [55].

#### 3.8.3. Trans-Anal Resection

TAR, which includes conventional open trans-anal excision, trans-anal endoscopic microsurgery (TEM) and trans-anal minimally invasive surgery (TAMIS), allows full thickness excision of rectal polyps that are suspected to be malignant or are too large to be excised using endoscopic techniques [7]. TEM and TAMIS are both minimally invasive techniques to resect lesions in the rectum that are too large for colonoscopic removal. Both TEM and TAMIS involves establishing pneumorectum using special trans-anal ports and the operation being carried out using minimally invasive instruments (conventional laparoscopic instruments for TAMIS). However, minimally invasive techniques are not possible if the lesion lies too distally or is too close to the dentate line [7]. In these cases, a conventional trans-anal excision technique will typically be used [7]. The key benefit of TAR is the ability to obtain a full thickness excision which enables the proper T staging of the malignant polyp. However, TAR still does not address any potential LNM [56].

#### 3.8.4. Role of Trans-Anal Excision Versus Total Mesorectal Excision (TME)

Due to these challenges with current staging modalities, TAR of the polypectomy site should be considered as part of a surgeon’s armamentarium when delineating the T stage of the malignant rectal polyp [48].

A Cochrane review published in 2022, which included four studies, found that local excision for Stage 1 rectal cancer (including both T1 and T2, node negative tumours) was associated with increased recurrence rates [57]. This, however, did not translate to an increase in cancer-related survival when compared to radical surgery. Satisfaction in bowel function and quality of life (QOL) was perceived to be better in the local excision group. An important drawback was the heterogeneity of the four studies which all had different co-interventions (use of neoadjuvant or adjuvant chemotherapy or radiotherapy) [57].

A German study found that patients who underwent TAR due to uncertain margins from an initial polypectomy had a local recurrence rate of 11% for low-risk malignant polyps and 25% for high-risk malignant polyps [58] on long-term follow-up post TAR. The median follow-up duration was over eight years in this study. The key finding was that none of these patients had residual cancer at the polypectomy site [58]. This raises the question regarding the duration of surveillance that should be recommended and whether regular surveillance with rectal MRI studies should be performed.

Whilst rectal resection with TME confers an excellent 5-year survival (87%) for early rectal cancer, it is also associated with a 20–30% risk of morbidity [42]. Proctectomy, especially that of the low rectum, is associated with high rates of low anterior resection syndrome (LARS) which impacts the patient’s quality of life [59]. Moreover, sphincter-preserving low-rectal resections are often associated with the use of a temporary ileostomy, which exposes patients to complications such as renal insufficiency and intestinal obstruction with resultant readmission rates as high as 25% [60]. In patients for whom sphincter preservation is not possible, decreased QOL associated with a permanent stoma is also not insignificant [60].

The most contemporary summary comparing local excision to TME in patients with T1/2 N0 rectal cancer is the network meta-analysis performed by Kwik et al. [59]. They found that despite a statistically significant decrease in local recurrence rate seen in the TME group, this did not translate into an improvement in the overall 5-year survival. The TME group was also associated with higher rates of 30-day mortality [59]. The authors, however, cautioned the quality of the included studies which only included three randomised controlled trials (27 observational studies).

It is also worth noting that Weiser et al. reported that salvage surgery for local failure post TAR was associated with a much poorer 5-year survival (50% vs. 90%) compared to patients who underwent upfront rectal resection with TME despite an initial clear microscopic margin resection [61]. It is, however, unclear if tumour characteristics were different between the two groups.

#### 3.8.5. Implications of Total Neoadjuvant Therapy (TNT) and Organ Preservation

With the advent of TNT, it has widely been accepted as the standard of care for managing locally advanced rectal cancer, popularised by the results of PRODIGE-23 and RAPIDO RCTs. The rates of pathological complete response (PCR) and disease-free survival was superior in the TNT cohort. As chemotherapy is given in the neoadjuvant setting, completion rates were also found to be higher compared to in the adjuvant setting [62].

The promising PCR rates also resulted in the increasing acceptance of the W&W approach with the aim of rectal preservation. The OPRA trial, the largest phase 2 RCT for W&W, reported that 50% of the patients enrolled into W&W were successful in avoiding TME. Most importantly, the cohort requiring subsequent surgery due to clinical recurrence did not perform worse compared to the upfront rectal resection with TME group [63]. It is important to highlight that W&W requires 4-monthly clinical, endoscopic and MRI assessment which can be labour and resource intensive [62]. Although the OPRA trial did not include any patients with Stage 1 rectal cancer, the promising PCR rates can likely be extrapolated. This is especially relevant in patients with very low rectal malignant polyps whereby a resection either results in a very low anastomosis with higher risk of LARS or an abdominoperineal resection leading to a permanent stoma [64].

However, at present, this approach is not standard of care. Moreover, consideration should be given to the potentially unnecessary toxicity associated with chemotherapy which could be avoided if upfront surgery was performed [62].

At the time of this review, societal guidelines do not recommend “W&W” as standard of care in management of rectal cancer [65]. The ongoing STAR-TREC trial, which is a multi-centre RCT comparing “W&W” to TME in patients with T1-3N0 rectal cancer who have undergone TNT, may shed light on the feasibility of “W&W” in the management of early rectal cancer [66].

## 4. Future Direction

Whilst the endoscopic features suggestive of malignant polyps and submucosal invasion are well validated, it still relies on the endoscopist’s ability to pick up these changes. The push for more minimalistic approach to malignant polyps will require accurate recognition which relates to the appropriate management endoscopically. With the penetration of artificial intelligence (AI) in endoscopy, with software for adenoma detection such as GI Genius^TM^ (Medtronic, Sydney, Australia) [67], AI prediction of submucosal invasion might not be far away.

Whilst studies and meta-analyses have identified histopathological and biomarkers that predict LNM, hence the need for surgical resection, a validated scoring system to aid risk prediction is lacking. Current practice relies on the surgeon’s interpretation of acceptable risk when discussing management with patients.

Future research into the utility of AI-assisted technologies and the development of risk calculators to predict the risk of LNM and local recurrence in endoscopically resected malignant polyps would help streamline its management. However, this will likely require large multi-centre collaboration to acquire a sufficient sample size.

## 5. Conclusions

The management of malignant colorectal polyps lies in risk-stratifying patients who will benefit from an oncological resection. Histopathological reports should clearly specify the margins, depth of invasion, tumour grade, LVI and tumour budding. If high-risk features are present, consideration should be given to surgical resection. Rectal malignant polyps should be considered differently compared to its colonic counterpart. A low threshold for utilising TAR to fully excise the polyp and to assess the margins should be considered. The implications of rectal resection with TME should be part of the patient discussion when formulating an individualised management approach in patients with low rectal malignant polyps.

## Figures and Tables

**Table 1:** Consensus molecular subtype. Adapted from Hassnoot et al., (2020) [33].

Consensus Molecular Subtype	Percentage of CRC	Biomarkers	Prognostic Implications
1	13%	Microsatellite instability (MSI) BRAF	Favourable in early CRC
2	37%	Activated WNT- and MYC-pathways Increased EGFR expression with mutated TP53 gene	-
2	13%	KRAS mutation	-
4	23%	Epithelial–mesenchymal transition TGF-beta pathway Stromal activation	Worse relapse free and overall survival
